# Comparative evaluation of non-invasive tests for risk stratification for cause specific mortality in at-risk population of hepatic fibrosis

**DOI:** 10.1038/s41598-024-56085-3

**Published:** 2024-03-26

**Authors:** Huiyul Park, Eileen L. Yoon, Mimi Kim, Hye-Lin Kim, Mi Kyung Kim, Yu-Mi Kim, Dae Won Jun

**Affiliations:** 1 Department of Family Medicine, Myoungji Hospital, Hanyang University College of Medicine, Seoul, Korea; 2 Department of Internal Medicine, Hanyang University College of Medicine, 222 Wangsimni-ro, Seongdong-gu, Seoul, 04763 Korea; 3 Hanyang Institute of Bioscience and Biotechnology, Hanyang University, Seoul, South Korea; 4 Department of Radiology, Hanyang University College of Medicine, Seoul, Korea; 5 College of Pharmacy, Sahmyook University, Seoul, Republic of Korea; 6 Department of Preventive Medicine, Hanyang University College of Medicine, 222 Wangsimni-ro, Seongdong-gu, Seoul, 04763 Republic of Korea; 7 Graduate School of Public Health, Hanyang University College of Medicine, 222 Wangsimni-ro, Seongdong-gu, Seoul, 04763 Republic of Korea

**Keywords:** Non-alcoholic fatty liver disease, Hepatic and extrahepatic mortality, At-risk population, Fibrosis-4 index, SAFE score, Gastroenterology, Hepatology

## Abstract

Our study aimed to conduct a comparative evaluation of various noninvasive tests (NITs) for risk stratification in at-risk population for non-alcoholic fatty liver disease (NAFLD), focusing on cardiovascular and liver-related mortality. A total of 21,715 adults aged 40 years and older were enrolled at baseline. The mean follow-up period was 12.39 years. Three types of NITs (fibrosis-4 index [FIB-4], NAFLD fibrosis score [NFS], and steatosis-associated fibrosis estimator [SAFE] score) were used. When using the low cut-off as a 'rule-out' strategy, there were no significant differences in cardiovascular mortality between the 'rule-out' (low-risk) group and the 'rule-in' (intermediate- or high-risk) group based on FIB-4 (aHR = 1.029, *P* = 0.845) or NFS (aHR = 0.839, *P* = 0.271) classification. However, the SAFE score exhibited higher sensitivity in predicting cardiovascular mortality compared to FIB-4 or NFS (73.3% in SAFE score vs. 29.6% in FIB-4 or 21.3% in NFS). Only the SAFE score could effectively differentiate the risk between low- and intermediate- or high-risk groups for all types of mortality (all *P* values for aHR < 0.001). The low cutoff value of the SAFE score discriminated not only liver-related mortality but also identified the cardiovascular high-risk group in the community cohort.

## Introduction

As the global burden of hepatic fibrosis continues to rise, there is a growing consensus among liver societies and guidelines to recommend screening for advanced hepatic fibrosis in specific patient groups^1^, such as those with obesity, chronically elevated liver enzymes, type 2 diabetes, and metabolic syndrome, as well as in individuals with fatty liver^2–5^. These patient groups are referred to as the 'at-risk population' for non-alcoholic fatty liver disease (NAFLD)-related fibrosis. Current guidelines advocate the use of fibrosis index-4 (FIB-4) or NAFLD fibrosis score (NFS) as initial screening tools to identify individuals at high risk of advanced hepatic fibrosis within this at-risk population^6,7^.

Indeed, the 'at-risk group' not only faces a higher risk of liver disease but also experiences extrahepatic adverse outcomes, including cardiovascular diseases and malignancies^8–10^. Therefore, it is crucial to develop a comprehensive risk assessment algorithm that can effectively evaluate the risk of mortality related to liver and cardiovascular diseases and extrahepatic malignancies. This approach enables a more holistic evaluation of the risk profile of at-risk individuals, thereby aiding in early detection and targeted interventions for better patient outcomes. Previous research has demonstrated that employing high cutoff values of FIB-4 and NFS tests can be effective in discriminating cardiovascular and overall mortality, in addition to assessing liver disease, in patients with NAFLD^11^. However, in the real-world, non-invasive tests (NITs) primarily utilize a low cut-off as a rule-out strategy for identifying high-risk groups. To date, there is a lack of data on whether a low cut-off of NITs, which are used as the first tier for screening advanced hepatic fibrosis in the general population, can provide a holistic evaluation of high-risk groups, including mortality rates due to cardiovascular diseases and other non-liver-related conditions.

The steatosis-associated Fibrosis Estimator (SAFE) score not only demonstrated superior diagnostic performance in identifying subjects with significant fibrosis compared to FIB-4 and NFS but also exhibited a strong correlation with overall mortality in the general population. These findings suggest that the SAFE score has the potential to effectively stratify populations at risk of hepatic fibrosis and predict adverse clinical outcomes, particularly in primary care settings or populations with a low prevalence of advanced hepatic fibrosis^12,13^. However, further validation in other ethnic groups and studies related to hepatic and extrahepatic outcomes are necessary to establish its broader applicability. Additionally, future investigations should focus on assessing the ability of NITs to evaluate the overall mortality risk in high-risk populations, considering cardiovascular diseases, extrahepatic malignancies, and liver disease. Such comprehensive assessments will aid better risk stratification and early intervention strategies for improved patient management.

To date, few studies have explored the efficacy of various NITs as first-tier screening tools to identify high-risk groups in community cohorts, particularly concerning liver-related and hard cardiovascular outcomes. Our study aimed to investigate whether a low NIT cutoff could offer a comprehensive assessment of both hepatic and cardiovascular hard outcomes in a community cohort with a low prevalence of liver fibrosis.

## Methods

### Characteristics of cohort

The Cardiovascular Disease Association Study (CAVAS) was established as a part of the Korea Genomic Epidemiology Study (KoGES) which is a nationwide prospective cohort study led by the Korea Disease Control and Preventive Agency (KDCA)^14^. The KoGES-CAVAS study included six rural areas: the Multi-Rural Communities cohort (MRCohort) in Yangpyeong, Namwon, and Goryeong; the ARIRANG in Wonju and Pyeongchang; and the Kangwha cohort. These three cohorts were initially separate but were later combined into the CAVAS with a standardized protocol starting in 2008. A total of 21,715 participants who provided written informed consent were recruited between January 2005 and December 2011. Further information on the research design can be found in a previous study^15^. This study was conducted in accordance with the Declaration of Helsinki and Istanbul and was approved by the Institutional Review Board of Hanyang University Hospital (IRB No. HY-2022-11-012).

### Follow up and mortality

Follow-up visits were conducted every 2–4 years from 2007 to 2017. The cause and time of death were determined as of December 2022 by linking the non-identifying information of the cohort patients with death statistics obtained from the Korea Statistical Information Service (KOSIS). Information on the cause of death was obtained from the ICD10-based diagnosis at the time of death. The causes of death, including cardiac, liver, and extrahepatic malignancies, and their matched ICD10 codes are detailed in Supplementary Table 1.

### Inclusion and exclusion criteria

**Figure 1:**
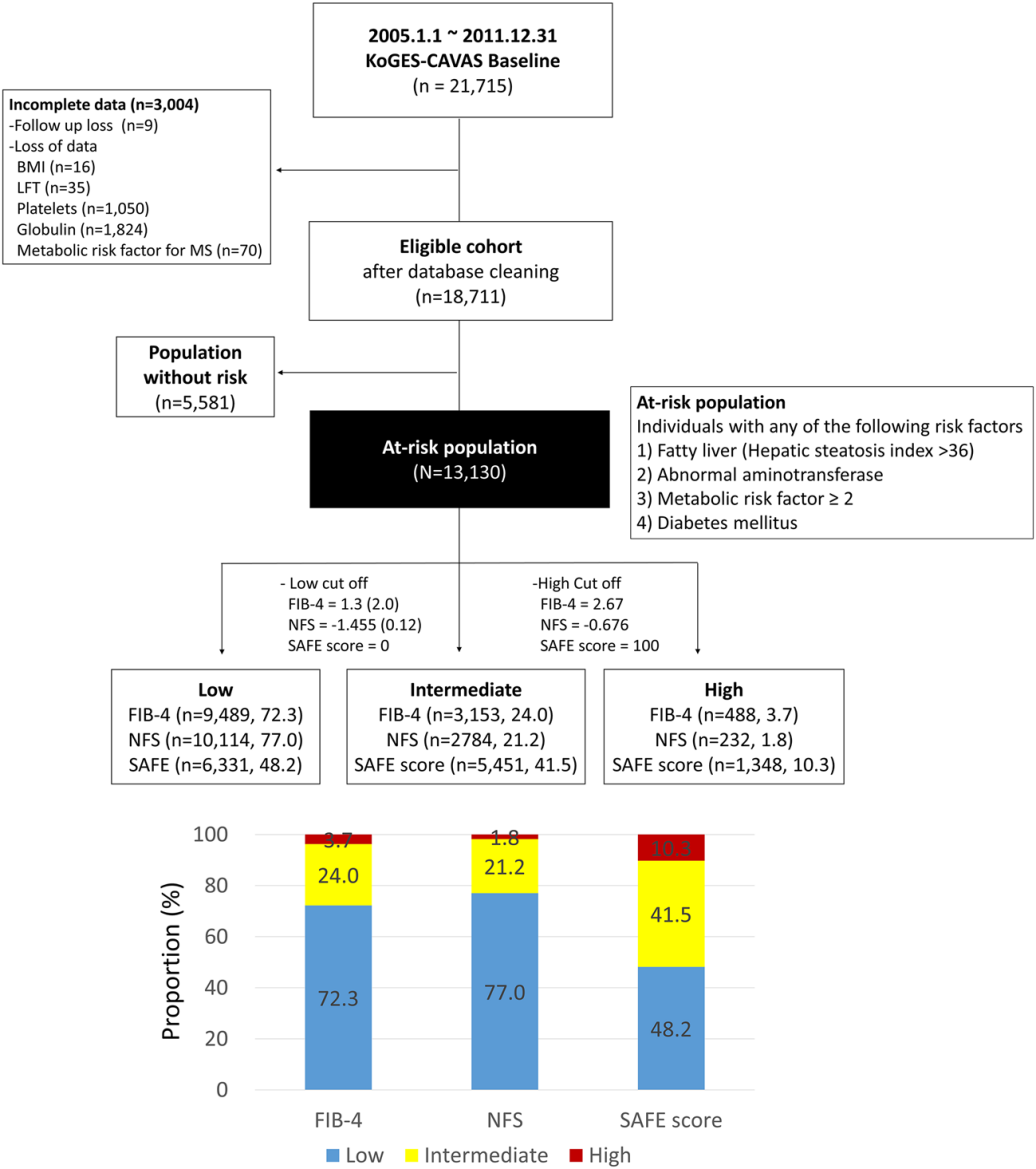
Study flowchart. *BMI* body mass index, *FIB-4* fibrosis-4 index, *LFT* liver function test, *SAFE* steatosis-associated fibrosis estimator.

The inclusion criteria were as follows: (1) 40 years at the beginning of the observation period. (2) Information on the ICD10-based diagnosis of death was available for all patients (Supplementary Table 1). (3) At least 1 year of medical history and 2 years of follow-up. The exclusion criteria were as follows: (1) loss to follow-up (n = 9). (2) Inappropriate information about BMI (n = 16), laboratory variables for calculating three noninvasive tests (NITs) (liver enzymes [n = 35], platelets [n = 1050], globulin [n = 1824]), and diagnosis of metabolic syndrome (n = 70) (Fig. 1).

### Target population of analysis

A total of 24,000 participants were included in the community-based rural cohorts. After exclusion based on the exclusion criteria, a total of 13,130 participants who had any risk factors such as fatty liver (hepatic steatosis index [HIS] > 36), two or more metabolic abnormalities for diagnosing metabolic syndrome, diabetes mellitus, and abnormal liver enzymes were selected as the at-risk population and analyzed. The mean follow-up was 12.4 years.

### Clinical parameters

History of hypertension, diabetes, or dyslipidemia, intake of corresponding medications for these conditions, and social history of alcohol drinking status were obtained from the questionnaires. Alcohol drinking status was categorized as non-, past or current drinker. The anthropometric measurements included waist circumference, blood pressure, height, weight, total fat mass, and lean mass. Additionally, fasting serum glucose, total cholesterol, low-density lipoprotein cholesterol, high-density lipoprotein cholesterol, triglycerides, total protein, albumin, AST, ALT, and-glutamyl transferase levels were measured.

### Calculation of NITs (SAFE score, FIB-4 and NFS)

FIB-4 and NFS were calculated, and their cut-off values were selected based on a study by McPherson et al.^16^. In subjects aged over 65 years, the low/high cut-off values of FIB-4 and NFS were 2.0/2.67 and 0.12/0.676, respectively. In subjects aged less than 65 years, the low/high cutoff values of FIB-4 and NFS were 1.3/2.67 and -1.455/0.676, respectively. The SAFE score was calculated, and the cut-off values were selected based on the study by Sripongpun et al.^12^ the low/high cut-off values of the SAFE score were 0/100. If the NIT score (FIB-4, NFS, and SAFE score) was lower than the low cutoff values, the subjects were classified into the low-risk group. If the score was between the low and high cut-off values, the subjects were assigned to the intermediate-risk group. If the score was higher than the high cut-off value, the subjects were assigned to the high-risk group.

### Disease definition of fatty liver, metabolic syndrome, and at-risk group

The ‘at risk group’ was defined as the group of individuals with any of the following risk factors: fatty liver, two or more metabolic abnormalities, diabetes mellitus, and abnormal liver function test (serum aspartate transaminase [AST] > 40 IU/L or serum alanine transaminase [ALT] > 40 IU/L)^3^. Metabolic risk factors for diagnosing subject with metabolic syndrome were defined as follows^17^: (1) waist circumference ≥ 85 cm for women and ≥ 90 cm for men, (2) blood pressure ≥ 130/85 mmHg and/or medication history of anti-hypertensive medications, (3) serum triglycerides ≥ 150 mg/dL, (4) high-density lipoprotein cholesterol < 50 mg/dL for women and < 40 mg/dL for men, and (5) fasting glucose level ≥ 100 mg/dL with HbA1c ≥ 5.7% and/or medication history of anti-diabetes medications. Metabolic syndrome was defined as the having three or more metabolic risk factors. This study highly focused on comparing the performance of various NITs for identifying individuals at high risk of various death especially within a community-based at-risk population for hepatic fibrosis beyond NAFLD patients. Unlike the typical approach of diagnosing NAFLD, this study considered all individuals with fatty liver as part of the at-risk population regardless of their alcohol drinking status.

The hepatic steatosis index was calculated to identify the patients with fatty liver disease^18^. Subjects with fatty liver were defined as those with a hepatic steatosis index > 36.

### Statistical analyses

Continuous and categorical variables are presented as mean ± standard deviation and numbers and percentages, respectively. Continuous variables were analyzed using the Student’s independent *t*-test, and categorical variables were analyzed using the chi-square test. The areas under the receiver operating characteristic curves (AUROC) of FIB-4, NFS, and SAFE scores for predicting clinical hard outcomes were compared using DeLong’s test in MedCalc (version 20; MedCalc Software Ltd., Ostend, Belgium). Sensitivity, specificity, positive predictive value (PPV), and negative predictive value (NPV) at low cutoff values were assessed. The incidence rates of various mortalities were calculated by dividing the total number of cases by the observation time (1000 person–years). 100-Survival probability (%) versus years of follow-up was generated using the Kaplan–Meier method. Univariate and multivariate Cox regression results for clinical outcomes such as all cause, cardiac, liver, and extrahepatic malignancy mortality according to grade of NITs were reported as hazard ratios (HR) and 95% confidence intervals (95% CI). Sex, presence of hypertension, high triglyceride levels, and low HDL levels were adjusted. The presence of diabetes was additionally adjusted for when estimating the adjusted HR for various deaths according to the grade of FIB-4. Statistical analyses were performed using SPSS software (version 26.0; IBM Corp., Armonk, NY, USA). Statistical significance was set at *P* < 0.05.

## Results

### Baseline characteristics of ‘at-risk population’

In total, 70.2% (13,130/18,711) of KoGES-CAVAS cohort was identified as ‘at-risk’ (Fig. 1). The prevalence of fatty liver, two or more metabolic abnormalities, diabetes mellitus, and abnormal liver function in these populations were 24.4%, 65.7%, 12.3%, and 29.7%, respectively (Table 1). Those in the ‘at risk group’ were older and had more unfavorable metabolic profiles, such as higher body mass index (BMI), waist circumference, blood pressure, and serum triglyceride and glucose levels, and higher liver enzymes, compared with those not at risk. When the low cutoffs of three NITs were used as the standard for further evaluation, 27.7% for FIB-4, 23.0% for NFS, and 51.8% for SAFE scores among at-risk populations needed the second step test.

**Table 1:** Clinical characteristics of community based rural cohort according to the presence of the risk for hepatic fibrosis.

Characteristics	Total subjects (n = 18,711)	Population without risk (n = 5581, 29.8%)	At-risk population (n = 13,130, 70.2%)	*P* value
Clinical characteristics at enrollment				
Age (years)^†^	58.4 ± 9.7	56.7 ± 10.2	59.2 ± 9.3	< 0.001
Male sex	7148 (38.2)	2152 (38.6)	4996 (38.1)	0.512
BMI (kg/m^2^)^†^	24.4 ± 3.1	22.4 ± 2.2	25.2 ± 3.0	< 0.001
Waist circumference (cm)^†^	84.1 ± 8.8	77.9 ± 6.8	86.7 ± 8.3	< 0.001
Systolic blood pressure (mmHg)^†^	124 ± 17	115 ± 15	127 ± 17	< 0.001
Glucose (mg/dL)^†^	98 ± 22	89 ± 7	102 ± 25	< 0.001
Triglyceride (mg/dL)^†^	147 ± 99	94 ± 34	170 ± 108	< 0.001
HDL (mg/dL)^†^	45 ± 11	51 ± 11	43 ± 10	< 0.001
Number of metabolic abnormality^†^	2.16 ± 1.33	0.65 ± 0.47	2.80 ± 1.04	< 0.001
Hypertension	8065 (43.1)	899 (16.1)	7166 (54.6)	< 0.001
Diabetes	2294 (12.3)	0 (0)	2294 (17.5)	< 0.001
Fatty liver (HIS > 36)	4563 (24.4)	0 (0)	4563 (34.8)	< 0.001
Metabolic abnormality ≥ 2	12,288 (65.7)	0 (0)	12,288 (93.6)	< 0.001
Metabolic syndrome	7440 (39.8)	0 (0)	7440 (56.7)	< 0.001
Abn. LFT	5558 (29.7)	0 (0)	5558 (42.3)	< 0.001
AST (IU/L)^†^	26 ± 22	23 ± 5	28 ± 26	< 0.001
ALT (IU/L)^†^	24 ± 18	18 ± 6	26 ± 21	< 0.001
Total protein (mg/dL)^†^	7.3 ± 0.4	7.3 ± 0.4	7.4 ± 0.4	< 0.001
Albumin (mg/dL)^†^	4.4 ± 0.2	4.4 ± 0.2	4.4 ± 0.2	< 0.001
Platelets (× 10^9^/L)^†^	257 ± 61	251 ± 58	260 ± 63	< 0.001
Alcohol drinking status				
Non-drinker	9793 (52.4)	2935 (52.7)	6858 (52.3)	0.023
Past drinker	1161 (6.2)	305 (5.5)	856 (6.5)	
Current drinker	7728 (41.4)	2331 (41.8)	5397 (41.2)	
Hepatic steatosis index^†^	33.1 ± 4.6	29.9 ± 2.9	34.5 ± 4.5	< 0.001
FIB-4 index^†^	1.37 ± 1.84	1.40 ± 3.03	1.37 ± 0.97	0.306
NFS^†^	− 1.94 ± 1.18	− 2.24 ± 1.01	− 1.81 ± 1.18	< 0.001
SAFE score^†^	− 1.3 ± 74.3	− 25.7 ± 63.1	8.9 ± 76.2	< 0.001
Clinical outcomes during follow-up period				
Overall death	2187 (11.7)	561 (10.1)	1626 (12.4)	< 0.001
Cardiac death	324 (1.7)	84 (1.5)	240 (1.8)	0.122
Liver death	129 (0.7)	15 (0.3)	114 (0.9)	< 0.001
Death from extrahepatic malignancy	636 (3.4)	181 (3.2)	455 (3.5)	0.443

Data are expressed as number (percent).

*Abn* abnormal, *ASCVD* atherosclerotic cardiovascular disease, *AST* aspartate transaminase, *ALT* alanine transaminase, *BMI* body mass index, *FIB-4 index* fibrosis-4 index, *HDL* high density lipoprotein, *HIS* hepatic steatosis index, *LFT* Liver function test, *NAFLD* non-alcoholic fatty liver disease, *NFS* NAFLD fibrosis score.

^†^Data are shown as mean ± standard deviation.

### Predictive value of cardiovascular mortality using low cut-off among various NITs

**Figure 2:**
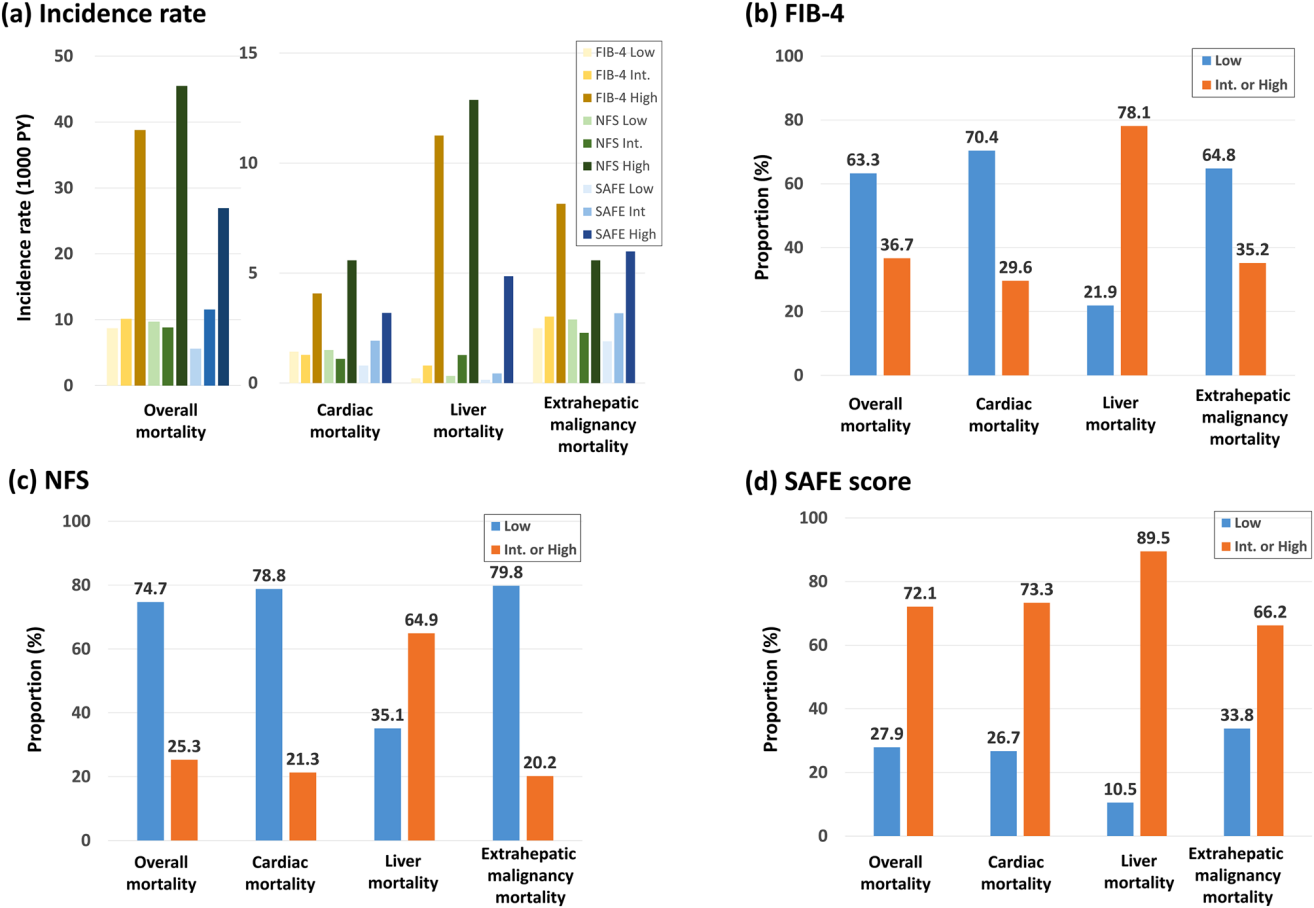
Hard outcome events during follow-up period. (**A**) Incidence rate (number of cases/1000 person-years) of overall mortality, cardiac mortality, liver mortality, and mortality from extrahepatic malignancy in at-risk population according to the ties of FIB-4, NFS, and SAFE score. The proportion of the low-risk and intermediate/high-risk groups identified by FIB-4 (**B**), NFS (**C**), and SAFE score (**D**) in various death. *FIB-4* fibrosis-4 index, *Int* intermediate, *NAFLD* nonalcoholic fatty liver disease, *NFS* NAFLD fibrosis score, *SAFE score* steatosis-associated fibrosis estimator score.

During a median follow-up of 12.3 years, 1626 of the 13,130 individuals in the at-risk population died (Table 1). Out of the total deaths, cardiovascular-related deaths accounted for 14.8% (240/1626), liver disease-related deaths for 7.0% (114/1626), and extrahepatic malignancy-related deaths were 28.0% (455/1626). The high-risk group selected by the high cutoff of all NITs (FIB-4, NFS, and SAFE score) showed the highest incidence of various mortality rates (cardiovascular, liver, and extrahepatic malignancy-related deaths). However, the intermediate-risk group selected between the low and high cutoff of FIB-4 and NFS did not show significant differences in the incidence rates of cardiovascular and extrahepatic malignancy-related deaths compared to those in the low-risk group (Fig. 2A). Only the SAFE classification showed a consistently increasing trend in incidence rates across all types of mortality.

### The low cutoff of the SAFE score can minimize the number of missed patients in all types of mortality

In practice, NITs are used to rule out or rule in risk assessments by applying a low cut-off value. Of the total deaths caused by liver problems, the proportion in the group below the low cutoff value (low-risk group) was lower than that in the group above the low cutoff value (intermediate- or high-risk group) for all NITs (Fig. 2B–D). However, the proportion of cardiovascular mortality belonging to the group below the low cutoff (low-risk group) was higher than that in the group above the low cutoff (intermediate- or high-risk group) for both FIB-4 (70.4% vs. 29.6%) and NFS (78.8% vs. 21.3%) (Fig. 2B,C). Similarly, the proportion of extrahepatic malignancy-related mortality belonging to the group below the low cutoff was also higher than in the group above the low cutoff for both FIB-4 (64.8% vs. 35.2%) and NFS (79.8% vs. 20.2%). Only in the case of the SAFE score, the proportion of cardiovascular (26.7% vs. 73.3%) and extrahepatic malignancy (33.8% vs. 66.2%)-related mortality belong to the group below the low cutoff were lower, compared to the group above the cut-off (Fig. 2D). The low cutoff values of both FIB-4 and NFS did not effectively distinguish the risk of all-cause, cardiovascular, and extrahepatic malignancy mortality between the 'rule-out' and 'rule-in' groups. However, the SAFE score consistently demonstrated a clear trend across all mortality types, with a higher number of deaths in the 'rule-in' group than in the 'rule-out' group.

### Diagnostic performance of three NITs for prediction of various mortalities

There were no differences in the AUROCs for predicting cardiac- and liver-related mortality among the three types of NITs (Table 2; Supplementary Table 2). When predicting overall mortality, AUROC was highest in order of FIB-4 (0.688, 95% CI 0.674–0.702), SAFE score (0.678, 95% CI 0.664–0.692), and NFS (0.659, 95% CI 0.645–0.674) (*P* values, FIB-4 vs. SAFE score: 0.026; FIB-4 vs. NFS: < 0.001; SAFE score vs. NFS: 0.001). All NITs showed a distinctively higher AUROC for predicting liver mortality than for predicting other mortalities. The SAFE score exhibited a higher sensitivity for predicting various types of mortality than the FIB-4 or NFS scores. Specifically, it demonstrated 2–3 times higher sensitivity for overall mortality (72.1% for SAFE score vs. 36.7% for FIB-4 or 25.3% for NFS) and cardiac mortality (73.3% for SAFE score vs. 29.6% for FIB-4 or 21.3% for NFS). Positive predictive value (PPV) and negative predictive value (NPV) were found to be comparable across all causes of death for the three types of NITs when applied low cut-off.

**Table 2:** Predictive ability of FIB-4, NFS and SAFE score for mortality due to various cause by using their low cut-off values in at-risk population.

Low cut-off	AUROCs (95% CI)	Sensitivity (95% CI)	Specificity (95% CI)	PPV (95% CI)	NPV (95% CI)	Accuracy (95% CI)
Overall mortality						
FIB-4 (> 1.3 (2.0))	0.688 (0.674–0.702)	36.7 (34.5–39.0)	73.5 (72.7–74.3)	16.4 (15.4–17.3)	89.1 (88.7–89.5)	68.9 (68.1–69.7)
NFS (− 1.455 (0.12))	0.659 (0.645–0.674)	25.3 (23.1–27.4)	77.4 (76.5–78.1)	13.6 (12.6–14.7)	88.0 (87.6–88.3)	70.9 (70.1–71.6)
SAFE score (> 0)	0.678 (0.664–0.692)	72.1 (69.8–74.3)	51.1 (50.1–52.0)	17.3 (16.7–17.7)	92.8 (92.2–93.3)	53.7 (52.8–54.5)
Cardiac mortality						
FIB-4 (> 1.3 (2.0))	0.650 (0.616–0.684)	29.6 (23.8–35.7)	72.3 (71.5–73.0)	2.0 (1.6–2.0)	98.2 (98.0–98.3)	71.5 (70.7–72.2)
NFS (− 1.455 (0.12))	0.626 (0.591–0.662)	21.3 (16.2–26.9)	77.0 (76.2–77.7)	1.7 (1.3–2.1)	98.1 (98.0–98.2)	75.9 (75.2–76.7)
SAFE score (> 0)	0.645 (0.609–0.680)	73.3 (67.2–78.8)	48.6 (47.7–49.4)	2.6 (2.3–2.7)	99.0 (98.7–99.1)	49.0 (48.2–49.9)
Liver mortality						
FIB-4 (> 1.3 (2.0))	0.847 (0.805–0.888)	78.1 (69.3–85.2)	72.7 (71.9–73.4)	2.4 (2.2–2.6)	99.7 (99.6–99.8)	72.7 (71.9–73.5)
NFS (− 1.455 (0.12))	0.834 (0.796–0.873)	64.9 (55.4–73.6)	77.4 (76.6–78.1)	2.5 (2.1–2.8)	99.6 (99.4–99.6)	77.2 (76.5–78.0)
SAFE score (> 0)	0.859 (0.818–0.900)	89.5 (82.3–94.4)	48.5 (47.6–49.4)	1.5 (1.4–1.6)	99.8 (99.6–99.8)	48.9 (48.0–49.7)
Extrahepatic malignancy mortality						
FIB-4 (> 1.3 (2.0))	0.647 (0.622–0.672)	35.2 (30.7–39.7)	72.5 (71.7–73.3)	4.4 (3.8–4.9)	96.9 (96.6–97.0)	71.2 (70.4–72.0)
NFS (− 1.455 (0.12))	0.602 (0.576–0.629)	20.2 (16.6–24.2)	76.9 (76.1–77.6)	3.1 (2.5–3.6)	96.4 (96.2–96.5)	74.9 (74.2–75.7)
SAFE score (> 0)	0.619 (0.593–0.644)	66.2 (61.6–70.4)	48.7 (47.8–49.6)	4.4 (4.1–4.7)	97.6 (97.2–97.8)	49.3 (48.4–50.1)

*AUROC* area under receiver operating characteristic, *CI* confidence interval, *FIB-4* fibrosis-4 index, *NAFLD* non-alcoholic fatty liver disease, *NFS* NAFLD fibrosis score, *NPV* negative predictive value, *PPV* positive predictive value, *SAFE score* steatosis-associated fibrosis estimator score.

### The risk assessments for various mortality by three NITs

**Figure 3:**
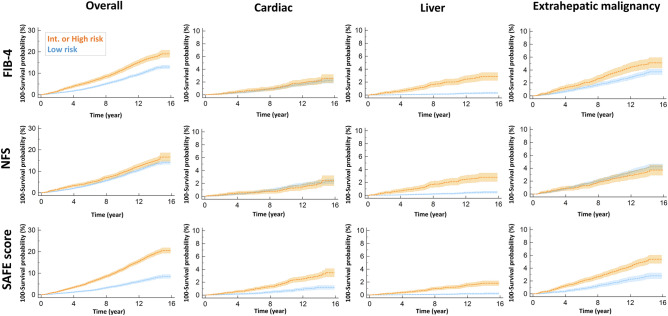
Mortality curve (100-survival probability) from overall, cardiac, liver, and extrahepatic malignancy according to tiers of three NITs. ‘100-survival probability’ versus years of follow-up graphs were generated by the Kaplan–Meier method. *FIB-4* fibrosis-4 index, *Int* intermediate, *NAFLD* nonalcoholic fatty liver disease, *NFS* NAFLD fibrosis score, *NITs* noninvasive tests, *SAFE score* steatosis-associated fibrosis estimator score.

**Figure 4:**
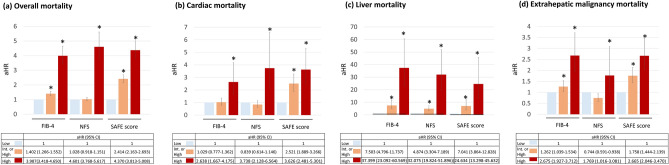
Adjusted hazard ratio (aHR) for various mortalities according to tiers of three NITs. Various mortalities include overall (**A**), cardiac (**B**), liver death (**C**), and death from extrahepatic malignancy (**D**). Statistical analyses were performed using Cox regression and results were reported as aHR and 95% CI. Sex, presence of hypertension, high triglyceride level, and low HDL level were adjusted in common. Presence of diabetes was additionally adjusted in case of estimating adjusted-HR for various death according to tiers of FIB-4. *CI* confidence interval, *FIB-4* fibrosis-4 index, *HR* hazard ratio, *Int* intermediate, *NAFLD* nonalcoholic fatty liver disease, *NFS* NAFLD fibrosis score, *NITs*, noninvasive tests, *SAFE score* steatosis-associated fibrosis estimator score.

The survival curve showed the highest mortality rate in individuals above the high cutoff, regardless of the NIT type or cause of death (Supplementary Fig. 1). However, when we utilized the low cut-off as a 'rule-out' strategy, as commonly practiced, there was no significant difference in cardiovascular mortality between the 'rule-out' (low-risk) group and the 'rule-in' (intermediate- or high-risk) group when using FIB-4 or NFS classification (Fig. 3). For NFS, no distinction in overall and extrahepatic malignancy mortality was observed between the ‘rule-out’ and ‘rule-in’ groups. Only the SAFE score could effectively differentiate between the low- and intermediate-risk groups across all types of mortality; there was no overlap area in survival curves between low-risk and intermediate- or high-risk groups. The results of both univariate and multivariate analyses consistently demonstrated that only the SAFE score was able to differentiate the risk of all kinds of mortalities between the low-risk ('rule-out') and intermediate- or high-risk ('rule-in') groups (all *P* values for HR < 0.001) (Supplementary Table 3, Fig. 4).

### The diagnostic performance of SAFE score in older populations

As individuals progressed from their 40 s to over 70 years of age, the proportion of those identified as the intermediate- or high-risk group (SAFE score > 0) also increased from 19.4 to 79.8% (Supplementary Table 4). In other words, approximately 80% of individuals over the age of 70 may be classified as positive for the test. Although the PPV increased with age (Supplementary Table 5), the absolute number of false positives increased only in older populations. Furthermore, the diagnostic performance of the SAFE score for overall mortality showed a low specificity and NPV of 20.8% and 64.1%, respectively, for those over 70 years of age (Supplementary Table 5). This finding suggests that the SAFE score has a lower ability to rule out clinical outcomes in older populations. In addition, the AUROCs for various mortality rates decreased with age. Taken together, the SAFE score showed low diagnostic performance for various mortalities in the older populations.

## Discussion

To the best of our knowledge, this is the first study to compare the performance of various NITs using a low cutoff value to identify individuals at high risk of cardiovascular and extrahepatic malignancy-related mortality within a community-based at-risk group, in addition to liver-related hard outcomes. The SAFE score appears to enable a more comprehensive evaluation of the risk profile of at-risk individuals for mortality related to liver, cardiovascular diseases, and extrahepatic malignancies. The FIB-4 and NFS scores demonstrated good predictive capabilities for cardiovascular and extrahepatic malignancy-related mortality when higher cut-off values were used. However, the low cut-off values for FIB-4 and NFS did not effectively distinguish cardiovascular mortality in the intermediate- or high-risk groups from that in the low-risk group. Considering that low cutoff values are commonly used in clinical practice for various noninvasive tests to exclude high-risk individuals^2–4^, the SAFE score exhibits an advantage over FIB-4 and NFS in the comprehensive evaluation and selection of high-risk groups with elevated risks of not only liver disease but also cardiovascular mortality within a community-based cohort. In terms of diagnostic performance, the AUROC values for predicting liver- and cardiovascular-related mortality were similar among the three NITs. This indicates that the superiority of the SAFE score lies in a reasonably low cutoff value for identifying high-risk individuals within a community-based cohort, rather than in its overall diagnostic performance in predicting cardiovascular and liver-related factors, compared to FIB-4 and NFS.

If the low cutoff values of FIB-4 and NFS were used to exclude advanced hepatic fibrosis within the community-based cohort, a significant percentage of deaths related to cardiovascular issues (70.4% for FIB-4 and 78.8% for NFS) would be overlooked (Fig. 2B,C). This means that individuals classified as having a low risk of FIB-4 and NSF could include subjects with a considerable risk of cardiovascular-related death, which was not captured by the low cut-off values. In contrast, the SAFE score consistently showed the superior predictability, more than 70% in sensitivity, for not only liver related but also overall and cardiovascular related deaths (Fig. 2D). Additionally, the low cut-off SAFE score showed comparable or better PPV in predicting various mortalities, despite the larger number of subjects diagnosed as positive by the SAFE score (Table 2). These findings imply that the SAFE score can be a more attractive option for the holistic evaluation of at-risk groups at the primary care level.

However, caution should be exercised when applying SAFE scores to older populations. Age is considered not only a significant risk factor for hepatic fibrosis and mortality but also a confounding factor that affects the accuracy of NITs. As age increases, so does the NIT score, which may lead to an overestimation. To address this issue, FIB-4 and NFS utilize higher cut-off values for individuals aged 65 years or older than for those in other age groups. A recent study also reported that the SAFE score demonstrated a lower ability to rule out clinically significant fibrosis in older populations (aged 60–80)^13^. Similar findings were also observed in our study, as mentioned in the Results section. Consequently, special caution will be required when applying the SAFE score to older populations.

The FIB-4 and NFS are primarily designed to screen for advanced hepatic fibrosis in patients with NAFLD or viral hepatitis^19,20^. In other words, the FIB-4 index and NFS were developed to screen for advanced hepatic fibrosis in high-risk patients. In contrast, the SAFE score was specifically developed to screen for significant hepatic fibrosis in populations with a low prevalence of advanced hepatic fibrosis, or in primary care settings. Because a considerable proportion of advanced fibrosis can be included in high-risk groups, such as patients with NAFLD or viral hepatitis, targeting advanced hepatic fibrosis as a screening strategy is a reasonable approach. However, in the case of the population or primary care settings, a small proportion of the overall population is compatible with advanced hepatic fibrosis. In this relatively benign population, targeting advanced hepatic fibrosis in the screening strategy is not a reasonable choice. Previous studies have also pointed out the above concerns regarding the appropriate target or cutoff values of NITs in a population with a low prevalence of advanced hepatic fibrosis^7,21^. Moreover, the overall or cardiovascular disease mortality in patients with NAFLD showed a dose-dependent relationship with the degree of hepatic fibrosis in previous studies^8,22,23^. From this perspective, the SAFE score, which includes individuals with significant hepatic fibrosis as diagnostic targets, can be used to predict mortality. Another reason is that the SAFE score includes additional metabolic parameters such as BMI and diabetes, which are associated with cardiovascular and overall mortality, making it more comprehensive than FIB-4.

This study has several limitations. First, hepatic steatosis index used as a diagnostic tool to assess fatty liver. This tool is an indirect method for evaluating the degree of steatosis in the liver, therefore it has lower accuracy than imaging tools for measuring the degree of steatosis directly, such as ultrasonography or MRI-PDFF. However, it also showed the good diagnostic performance for finding patients with NAFLD at higher cutoff values (specificity, 93.1%; PPV 86.7%)^18^. It can be also a more appropriate option for assessing fatty liver disease in primary care settings since its calculation variables (BMI, AST, ALT, presence of DM) are commonly used in such clinical settings. Moreover, our study focused on evaluating the predictive ability of the three NITs for long-term clinical outcomes in a heterogeneous group consisting of people with obesity, chronically elevated liver enzymes, type 2 diabetes, or metabolic syndromes other than fatty liver. There is a high probability that there will be few people with fatty liver disease without other risk factors. Therefore, we believe that the overall results did not differ. Second, almost all the participants were indwellers in rural areas. Therefore, the proportion of elderly people is high. Additionally, there was no consideration of the medication or underlying diseases of the participants owing to the design of the study. However, our cohort also has unique strengths. The cohort consisted of volunteers from six rural communities in South Korea. Local residents participated throughout South Korea, therefore it is expected to capture a more generalized picture of health and disease than a clinical disease-focused or health-screening cohort. The cohort size represented a substantial percentage (ranging to 4–10%) of the local population. The Epidemiological Data and Quality Management Center, which is responsible for overseeing the cohort centers in these communities, ensured the quality of data collection for this multicenter study, resulting in standardized and reliable data. Nevertheless, further study designed as prospective large-scale studies are required.

In conclusion, our results consistently showed that a low cut-off SAFE score could differentiate the risk of overall mortality, cardiovascular mortality, and mortality from extrahepatic malignancy between the low- and intermediate- or high-risk groups. It outperformed the low cutoff values of FIB-4 and NFS, particularly in predicting outcomes other than liver-related mortality. The low cutoff SAFE score allowed for holistic evaluation of both hepatic and extrahepatic high-risk groups within the community cohort. By utilizing the SAFE score with a low cutoff, it is possible to identify individuals who are at an elevated risk for both liver- and non-liver-related adverse outcomes, providing a comprehensive assessment of their overall health status. This approach ensures that groups at high risk for various health conditions are not overlooked, leading to more effective preventive strategies and interventions.

### Supplementary Information

Supplementary Information.

## Data Availability

The datasets generated and/or analyzed during this study are available from the corresponding authors on reasonable request.

## Abbreviations

ALT: Alanine aminotransferase
AST: Aspartate aminotransferase
AUROC: Area under the receiver operating characteristic curve
BMI: Body mass index
DM: Diabetes mellitus
FIB-4: Fibrosis-4 index
NAFLD: Nonalcoholic fatty liver disease
NFS: NAFLD fibrosis score
NPV: Negative predictive value
PPV: Positive predictive value
ROC: Receiver operating characteristic
