# Supplementary material for: Comparative evaluation of non-invasive tests for risk stratification for cause specific mortality in at-risk population of hepatic fibrosis
Source: Sci Rep. 2024 Mar 26;14:7189. doi: 10.1038/s41598-024-56085-3 (PMC10965918; doi:10.1038/s41598-024-56085-3)
Supplement: Supplementary file 1 — Supplementary Information. [file 41598_2024_56085_MOESM1_ESM.docx]

**Comparative Evaluation of Non-Invasive Tests for Risk Stratification for Cause Specific Mortality in At-risk Population of Hepatic Fibrosis**

Huiyul Park, Eileen L. Yoon, Mimi Kim, Hye-Lin Kim, Mi Kyung Kim, Yu-Mi Kim, and Dae Won Jun

**List of supplementary materials**

**1) Supplementary Table 1.** The definition of cause of death according to ICD10 code.

**2) Supplementary Table 2.** Comparison of AUROCs for various mortalities among three NITs.

**3) Supplementary Table 3.** Univariate and multivariate analyses of three NITs for various mortalities.

**4) Supplementary Table 4.** Proportion of subgroups classified by SAFE score according to age.

**5) Supplementary Table 5**. Predictive ability of SAFE score for mortality due to various cause by using their low cut-off values according to age

**6) Supplementary Figure 1.** Mortality curve (100-survival probability) from overall, cardiac, liver, and extrahepatic malignancy according to tiers (low-, int. -, and high-risk) of three NITs

| **Supplementary Table 1.** The definition of cause of death according to ICD10 code | |
| --- | --- |
| Cause of death | ICD10 code |
| Cardiac disease | "I10", "I11", "I12", "I13", "I14", "I15", or "I20", "I21", "I21", "I23", "I24", "I25", or "I60", "I61", "I62", "I63", "I64", "I65", "I66", "I67", "I68", "I69", or "I95", "I96", "I97", "I98", or "I99" |
| Liver disease | B15, "B16", "B17", "B18", "B19", or "C22", "K70", "K71", "K72", "K73", "K74", "K75", or  "K76", "K77", "K78", or "K79" |
| Extrahepatic malignancy | All codes starting "C", but except "C22" |

| **Supplementary Table 2.** Comparison of AUROCs for various mortalities among three NITs. | | | | | | | |
| --- | --- | --- | --- | --- | --- | --- | --- |
| AUROCs (95% CI) | NITs | | |  | *P* value | | |
|  | A) FIB-4 | B) NFS | C) SAFE score |  | A vs B | A vs C | B vs C |
| Overall mortality | 0.688 (0.674-0.702) | 0.659 (0.645-0.674) | 0.678 (0.664-0.692) |  | <0.001 | 0.026 | 0.001 |
| Cardiac mortality | 0.650 (0.616-0.684) | 0.626 (0.591-0.662) | 0.645 (0.609-0.680) |  | 0.093 | 0.622 | 0.215 |
| Liver mortality | 0.847 (0.805-0.888) | 0.834 (0.796-0.873) | 0.859 (0.818-0.900) |  | 0.473 | 0.283 | 0.145 |
| Extrahepatic malignancy mortality | 0.647 (0.622-0.672) | 0.602 (0.576-0.629) | 0.619 (0.593-0.644) |  | <0.001 | 0.001 | 0.127 |
| *P* value for the difference in AUROCs among three NITs was evaluated by DeLong’s test Abbreviations: AUROC, area under receiver operating characteristic; CI, confidence interval; FIB-4, fibrosis-4 index; NAFLD, non-alcoholic fatty liver disease; NFS, NAFLD fibrosis score; NITs, non-invasive tests; NPV, negative predictive value; PPV, positive predictive value. SAFE score, steatosis-associated fibrosis estimator score. | | | | | | | |

| **Supplementary Table 3**. Univariate and multivariate analyses of three NITs for various mortalities. | | | | | | | | | | | |
| --- | --- | --- | --- | --- | --- | --- | --- | --- | --- | --- | --- |
| **A) FIB-4** | Overall | |  | Cardiac | |  | Liver | |  | Extrahepatic malignancy | |
| Unadjusted HR | HR (95% CI) | *P* value |  | HR (95% CI) | *P* value |  | HR (95% CI) | *P* value |  | HR (95% CI) | *P* value |
| Low | 1 (Ref.) |  |  | 1 (Ref.) |  |  | 1 (Ref.) |  |  | 1 (Ref.) |  |
| Int. | 1.162 (1.035-1.305) | 0.011 |  | 0.895 (0.653-1.228) | 0.493 |  | 3.752 (2.215-6.354) | <0.001 |  | 1.210 (0.978-1.499) | 0.080 |
| High | 4.647 (3.994-5.408) | <0.001 |  | 2.964 (1.883-4.666) | <0.001 |  | 54.273 (33.942-86.781) | <0.001 |  | 3.343 (2.419-4.620) | <0.001 |
| Int. or High | 1.553 (1.404-1.718) | <0.001 |  | 1.128 (0.855-1.489) | 0.393 |  | 9.535 (6.118-14.859) | <0.001 |  | 1.454 (1.199-1.762) | <0.001 |
|  | Overall | |  | Cardiac | |  | Liver | |  | Extrahepatic malignancy | |
| aHR* | aHR (95% CI) | *P* value |  | aHR (95% CI) | *P* value |  | aHR (95% CI) | *P* value |  | aHR (95% CI) | *P* value |
| Low | 1 (Ref.) |  |  | 1 (Ref.) |  |  | 1 (Ref.) |  |  | 1 (Ref.) |  |
| Int. | 1.062 (0.945-1.194) | 0.312 |  | 0.817 (0.594-1.122) | 0.212 |  | 3.138 (1.847-5.330) | <0.001 |  | 1.067 (0.860-1.323) | 0.557 |
| High | 3.987(3.418-4.650) | <0.001 |  | 2.638 (1.667-4.175) | <0.001 |  | 37.399 (23.092-60.569) | <0.001 |  | 2.675 (1.927-3.712) | <0.001 |
| Int. or High | 1.402 (1.266-1.552) | <0.001 |  | 1.029 (0.777-1.362) | 0.845 |  | 7.503 (4.796-11.737) | <0.001 |  | 1.262 (1.039-1.534) | 0.019 |
| **B) NFS** | Overall | |  | Cardiac | |  | Liver | |  | Extrahepatic malignancy | |
| Unadjusted HR | HR (95% CI) | *P* value |  | HR (95% CI) | *P* value |  | HR (95% CI) | *P* value |  | HR (95% CI) | *P* value |
| Low | 1 (Ref.) |  |  | 1 (Ref.) |  |  | 1 (Ref.) |  |  | 1 (Ref.) |  |
| Int. | 0.912 (0.805-1.034) | 0.152 |  | 0.732 (0.517-1.037) | 0.079 |  | 3.990 (2.600-6.122) | <0.001 |  | 0.790 (0.620-1.008) | 0.058 |
| High | 4.993 (4.093-6.091) | <0.001 |  | 3.909 (2.227-6.806) | <0.001 |  | 41.529 (25.842-66.738) | <0.001 |  | 1.993 (1.146-3.466) | 0.015 |
| Int. or High | 1.156 (1.034-1.293) | 0.011 |  | 0.923 (0.678-1.258) | 0.612 |  | 6.296 (4.285-9.249) | <0.001 |  | 0.864 (0.687-1.086) | 0.210 |
|  | Overall | |  | Cardiac | |  | Liver | |  | Extrahepatic malignancy | |
| aHR† | aHR (95% CI) | *P* value |  | aHR (95% CI) | *P* value |  | aHR (95% CI) | *P* value |  | aHR (95% CI) | *P* value |
| Low | 1 (Ref.) |  |  | 1 (Ref.) |  |  | 1 (Ref.) |  |  | 1 (Ref.) |  |
| Int. | 0.809 (0.713-0.918) | 0.001 |  | 0.661 (0.465-0.938) | 0.021 |  | 3.110 (2.020-4.788) | <0.001 |  | 0.680 (0.532-0.868) | 0.002 |
| High | 4.601 (3.768-5.617) | <0.001 |  | 3.738 (2.128-6.564) | <0.001 |  | 32.075 (19.824-51.896) | <0.001 |  | 1.769 (1.016-3.081) | 0.044 |
| Int. or High | 1.028 (0.918-1.151) | 0.637 |  | 0.839 (0.614-1.146) | 0.271 |  | 4.874 (3.304-7.189) | <0.001 |  | 0.744 (0.591-0.938) | 0.012 |
| **C) SAFE score** | Overall | |  | Cardiac | |  | Liver | |  | Extrahepatic malignancy | |
| Unadjusted HR | HR (95% CI) | *P* value |  | HR (95% CI) | *P* value |  | HR (95% CI) | *P* value |  | HR (95% CI) | *P* value |
| Low | 1 (Ref.) |  |  | 1 (Ref.) |  |  | 1 (Ref.) |  |  | 1 (Ref.) |  |
| Int. | 2.100 (1.869-2.358) | <0.001 |  | 2.473 (1.831-3.338) | <0.001 |  | 2.964 (1.512-5.808) | 0.002 |  | 1.683 (1.368-2.072) | <0.001 |
| High | 5.035 (4.402-5.758) | <0.001 |  | 4.200 (2.888-6.109) | <0.001 |  | 33.457 (18.160-61.637) | <0.001 |  | 3.223 (2.484-4.181) | <0.001 |
| Int. or High | 2.627 (2.357-2.928) | <0.001 |  | 2.784 (2.091-3.707) | <0.001 |  | 8.503 (4.674-15.466) | <0.001 |  | 1.963 (1.617-2.384) | <0.001 |
|  | Overall | |  | Cardiac | |  | Liver | |  | Extrahepatic malignancy | |
| aHR† | aHR (95% CI) | *P* value |  | aHR (95% CI) | *P* value |  | aHR (95% CI) | *P* value |  | aHR (95% CI) | *P* value |
| Low | 1 (Ref.) |  |  | 1 (Ref.) |  |  | 1 (Ref.) |  |  | 1 (Ref.) |  |
| Int. | 1.953 (1.753-2.214) | <0.001 |  | 2.273 (1.680-3.075) | <0.001 |  | 2.602 (1.326-5.105) | 0.005 |  | 1.543 (1.251-1.902) | <0.001 |
| High | 4.370 (3.813-5.008) | <0.001 |  | 3.626 (2.481-5.301) | <0.001 |  | 24.634 (13.298-45.632) | <0.001 |  | 2.665 (2.046-3.470) | <0.001 |
| Int. or High | 2.414 (2.163-2.693) | <0.001 |  | 2.521 (1.889-3.266) | <0.001 |  | 7.041 (3.864-12.828) | <0.001 |  | 1.758 (1.444-2.139) | <0.001 |
| Statistical analyses were performed using Cox regression and results were reported as HR and 95% CI. aHR* adjusted for Sex, presence of hypertension, type 2 diabetes, high triglyceride level, and Low HDL. aHR† adjusted for Sex, presence of hypertension, high triglyceride level, and Low HDL. Abbreviations: CI, confidence interval; FIB-4, fibrosis-4 index; HR, hazard ratio; NAFLD, non-alcoholic fatty liver disease; NFS, NAFLD fibrosis score; NITs, non-invasive tests; SAFE score, steatosis-associated fibrosis estimator score. | | | | | | | | | | | |

| **Supplementary Table 4.** Proportion of subgroups classified by SAFE score according to age. | | | | |
| --- | --- | --- | --- | --- |
| Subgroups | 40s n=2316 (17.6) | 50s n=4310 (32.8) | 60s n=4705 (35.8) | 70~ n=1799 (13.7) |
| Low | 1866 (80.6) | 2539 (58.9) | 1563 (20.2) | 363 (20.2) |
| Int. | 400 (17.3) | 1503 (34.9) | 2521 (53.6) | 1027 (57.1) |
| High | 50 (2.2) | 268 (6.2) | 621 (13.2) | 409 (22.7) |
| Int. or High | 450 (19.4) | 1771 (41.1) | 3142 (66.8) | 1436 (79.8) |

Data are expressed as number (percent).

| **Supplementary Table 5**. Predictive ability of SAFE score for mortality due to various cause by using their low cut-off values according to age | | | | | | |
| --- | --- | --- | --- | --- | --- | --- |
| SAFE score ( >0) | AUROCs (95% CI) | Sensitivity (95% CI) | Specificity (95% CI) | PPV (95% CI) | NPV (95% CI) | Accuracy (95% CI) |
| Overall mortality  40-59  60s  70~ | 0.617 (0.581-0.654) 0.592 (0.567-0.617) 0.560 (0.532-0.588) | 47.0 (41.0-53.1) 73.3 (69.8-76.6) 80.9 (77.8-83.8) | 67.0 (65.8-68.2) 34.3 (32.8-35.7) 20.8 (18.5-23.3) | 5.7 (5.0-6.5) 15.6 (15.0-16.3) 38.5 (37.3-39.6) | 96.7 (96.3-97.0) 88.5 (87.1-89.8) 64.1 (59.6-68.4) | 66.2 (65.0-67.3) 39.8 (38.4-41.2) 43.6 (41.3-46.0) |
| Cardiac mortality  40-59  60s  70~ | 0.607 (0.501-0.712) 0.574 (0.510-0.639) 0.480 (0.426-0.535) | 47.0 (29.7-64.8) 73.8 (63.4-82.6) 80.5 (72.2-87.2) | 66.5 (65.3-67.6) 33.3 (31.9-34.7) 20.2 (18.3-22.2) | 0.7 (0.5-1.0) 2.0 (97.9-98.9) 6.6 (6.0-7.2) | 99.5 (99.4-99.7) 98.5 (97.9-98.9) 93.6 (91.0-95.5) | 66.4 (65.2-67.5) 34.1 (32.7-35.4) 24.1 (22.1-26.2) |
| Liver mortality  40-59  60s  70~ | 0.843 (0.766-0.920) 0.867 (0.809-0.926) 0.819 (0.720-0.918) | 80.5 (63.9-91.8) 94.0 (83.4-98.7) 92.8 (76.4-99.1) | 66.7 (65.5-67.8) 33.5 (32.1-34.8) 20.4 (18.6-22.4) | 1.3 (1.1-1.53) 1.4 (1.3-1.6) 1.8 (1.6-2.0) | 99.8 (99.6-99.9) 99.8 (99.4-99.9) 99.4 (97.9-99.8) | 66.8 (65.6-67.9) 34.1 (32.8-35.5) 21.6 (19.7-23.6) |
| Extrahepatic malignancy mortality  40-59  60s  70~ | 0.576 (0.516-0.635) 0.528 (0.485-0.570) 0.515 (0.468-0.561) | 43.6 (33.3-54.2) 65.4 (58.3-72.0) 79.8 (72.9-85.7) | 66.6 (65.4-67.7) 33.1 (31.7-34.5) 20.1 (18.2-22.2) | 1.8 (1.4-2.3) 4.1 (3.6-4.7) 9.1 (8.4-9.8) | 98.7 (98.5-98.9) 95.6 (94.7-96.3) 90.9 (87.8-93.2) | 66.2 (65.1-67.4) 34.5 (33.1-35.8) 25.6 (23.6-27.7) |
| Abbreviations: AUROC, area under receiver operating characteristic; CI, confidence interval; NPV, negative predictive value; PPV, positive predictive value. SAFE score, steatosis-associated fibrosis estimator score. | | | | | | |


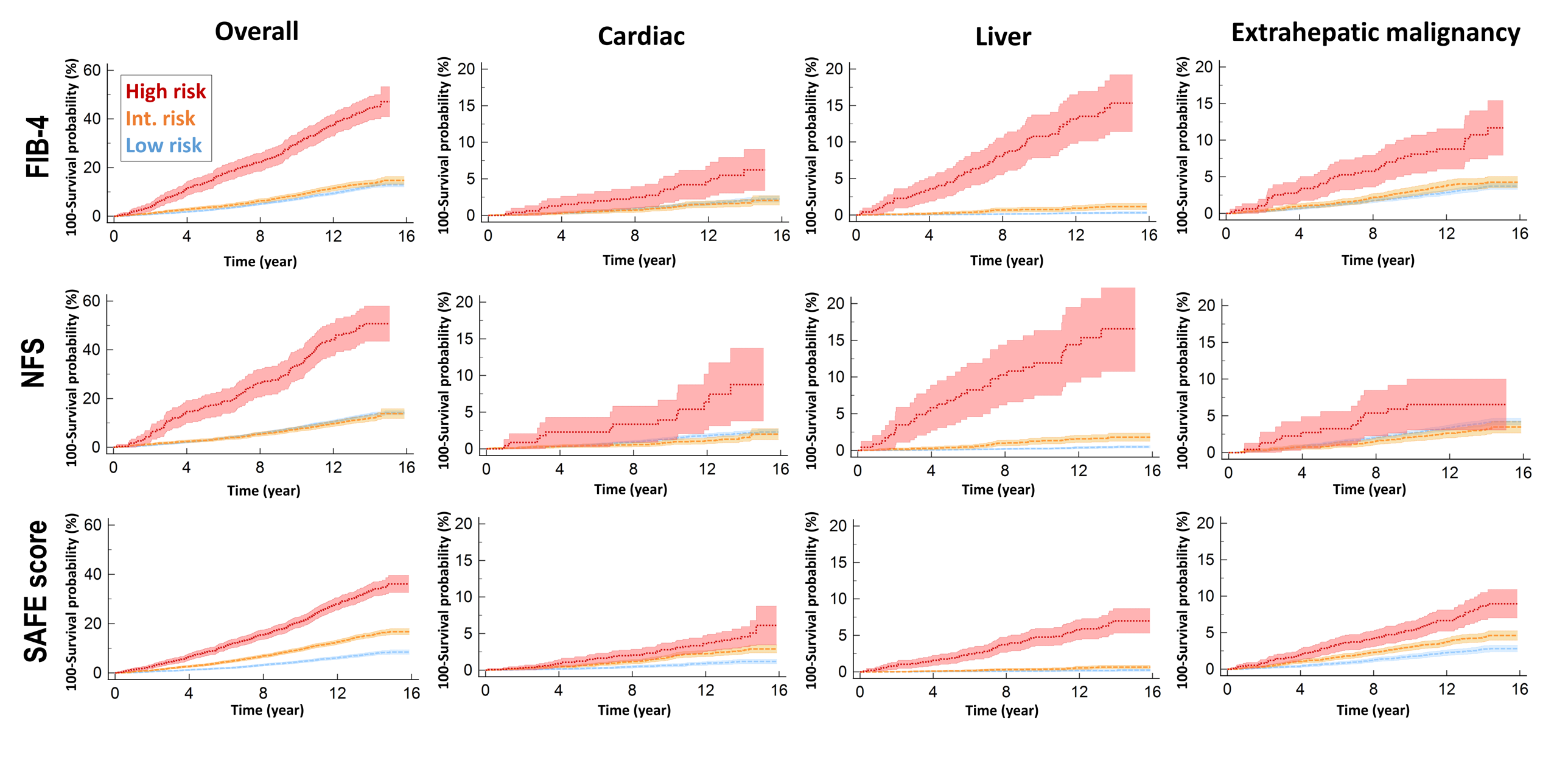


**Supplementary Figure 1.** Mortality curve (100-survival probability) from overall, cardiac, liver, and extrahepatic malignancy according to tiers (low-, int. -, and high-risk) of three NITs. `100-survival probability` versus years of follow-up graphs were generated by the Kaplan-Meier method.

Abbreviations: FIB-4, fibrosis-4 index; Int, intermediate; NAFLD, nonalcoholic fatty liver disease; NFS, NAFLD fibrosis score; NITs, noninvasive tests; SAFE score, steatosis-associated fibrosis estimator score.
